# Esophageal Papillomas as an Endoscopic Finding

**DOI:** 10.5152/eurasianjmed.2022.21056

**Published:** 2022-02-01

**Authors:** Juan Miguel Pacheco Bolaños, María Andrés Calvo, Esteban Fuentes-Valenzuela, Pablo Curiel Martínez, José Pablo Miramontes González

**Affiliations:** 1 Centro de Salud Parque Alameda-Covaresa-Valladolid, Spain; 2 Servicio de Anatomía Patológica. H. Universitario Río Hoterga, Valladolid, Spain; 3 Servicio de Aparato Digestivo. H. Universitario Río Hoterga, Valladolid, Spain; 4 Servicio de M. Interna. H. Universitario Río Hoterga. IBSAL- Instituto de Investigaciones Biomédicas de Salamanca. Facultad de Medicina – Universidad de Valladolid, Spain

**Dear Editor**

We present the clinical case of a 41-year-old woman with a medical history of iron deficiency anemia. The patient came to the consultation for abdominal pain, located in the epigastrium, and an alteration of the intestinal rhythm of 9 months evolution, referring to tenesmus and occasional hematochezia. An analysis was carried out (hemogram, coagulation, biochemistry, and autoimmunity study); low ferritin (4.8 ng/mL) was observed, with the rest of the study normal. To complete the study, abdominal ultrasound, gastroscopy, and colonoscopy were requested.

The gastroscopy revealed 2 hairy lesions in the esophagus 20-25 cm from the dental arch: these lesions were 2-3 mm in diameter each and had an appearance suggestive of papillomas (Figure 1A). These were excised and sent to pathological anatomy (Figure 1B), corroborating the diagnosis of esophageal papillomas. In the stomach, pylorus, and first and second duodenal portions, no alterations were observed.

## Discussion

Esophageal papilloma is an infrequent entity, with an approximate incidence that ranges between 0.01% and 0.04% in the population. They are seen more frequently in patients with tylosis and acanthosis nigricans, and there is an association with Goltz syndrome, a congenital disorder of focal dermal hypoplasia characterized by hyperpigmentation, sclerodactyly, dysplastic changes in teeth and bones, and papillomas in the perianal, oroesophageal, and genital regions.^1^

Pathogenesis is uncertain, although there are 2 proposed theories: the first is the inflammatory theory. Approximately 70% of papillomas occur in the distal third of the esophagus and have been associated with reflux, esophagitis, or irritants of the mucosa.^2^ The second theory relates these lesions to HPV infection. Studies carried out in populations of Italy and Hungary have documented the existence of HPV in 26-41% of esophageal papillomas.^3^ However, other studies in populations of the United States, Finland, and Poland have identified HPV in less than 5% of cases.^4^ The role of HPV in pathogenesis is supported by the fact that the most frequently found serotypes are 6 and 11 (the most frequent in the oropharynx and human genital tract). The route of acquisition of this viral infection is most often oral sex, but it has been considered possible via the ingestion of cellular particles from warty lesions from the hands and genitals.^5^ The evolution of these lesions to malignant forms is not frequent, although in cases with other concomitant risk factors, this evolution can be seen.

**Figure 1:**
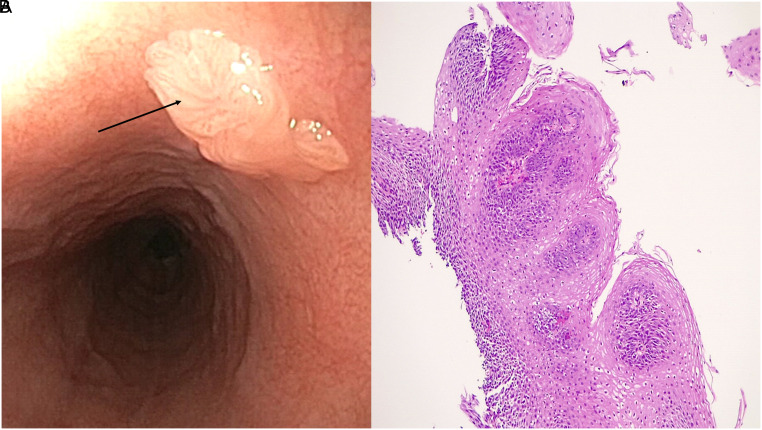
.(A) Endoscopic image of esophageal papillomas at the level of the esophagus. An exophytic lesion of vegetative appearance, with well-defined borders. (B) Pathological image of the esophageal papilloma with hematoxylin–eosin stain in which the classic structure of the papilloma can be seen. Mature stratified flat epithelium can be seen, with acanthosis and papillomatosis.

Diagnosis is usually by chance finding since they are mostly asymptomatic, but long-standing lesions can cause dysphagia if they are of considerable size. The macroscopic appearance on endoscopy is of small, whitish-pink exophytic projections, similar to warts, as seen in Figure 1A. The biopsy and the pathological study of the sample are considered essential for the diagnosis: a fibrovascular nucleus is classically observed, branching from the lamina propria to form finger-shaped projections without invasion toward the submucosa, completely surrounded by marked infiltration of neutrophils and covered by an acanthotic squamous epithelium,^10^ as seen in the image of the case study described in Figure 1B.
